# Exploring the Association Between COVID-19 and Avascular Necrosis: A Systematic Review

**DOI:** 10.7759/cureus.89318

**Published:** 2025-08-04

**Authors:** Mohamed Zahed, Alzahraa Faris Alesawy, Ziad Samir Zahed, Rahafat Samir, Mahmoud Eleisawy

**Affiliations:** 1 Orthopedics, John Radcliffe Hospital, Oxford University Hospitals NHS Trust, Oxford, GBR; 2 Clinical Microbiology and Immunology, Faculty of Medicine, Benha University, Benha, EGY; 3 Ophthalmology, Faculty of Medicine, Benha University, Benha, EGY; 4 Ophthalmology, Benha University Hospitals, Benha University, Benha, EGY

**Keywords:** avascular necrosis, corticosteroids, covid-19, osteonecrosis, systematic review

## Abstract

Avascular necrosis (AVN) has emerged as an extrapulmonary complication associated with COVID-19 and corticosteroids. This review aims to evaluate the association between COVID-19 infection, corticosteroid use, and the development of AVN. We conducted a systematic review following the PRISMA guidelines, searching five databases until May 30, 2024. We included cohort and case series studies involving COVID-19 patients who developed AVN. The risk of bias was assessed using the Newcastle-Ottawa Scale (NOS). A total of 13 studies, comprising nine case series and four cohort studies, were included. These studies involved 795 patients with a mean age of 46.1 years and a male predominance (66%). The cumulative dose of corticosteroids varied, with an average of 1,462.9 mg. The duration between COVID-19 infection and initial AVN symptoms ranged from 2 to 62 weeks. The most commonly affected bones were the hip and femoral head. The visual analog scale (VAS) score improved with the treatment, and the cases showed improvements. A significant association was found between COVID-19, corticosteroid use, and AVN development. Clinicians should exercise caution when prescribing corticosteroids and monitor for early signs of AVN. Further research is needed to elucidate the pathophysiological mechanisms and explore alternative treatments to mitigate the risk of AVN.

## Introduction and background

The coronavirus disease 2019 (COVID-19) pandemic, caused by the severe acute respiratory syndrome coronavirus 2 (SARS-CoV-2), has led to over 775 million infections and more than 7 million deaths globally [1]. The virus exerts direct cytopathic effects, particularly on the alveolar epithelium, but other organs, such as the liver and heart, can also be affected [2,3]. Additionally, a dysregulated immune response, characterized by the release of cytokines, can trigger a cytokine storm, potentially resulting in organ failure and death [4,5].

COVID-19 symptoms vary widely, including fever, cough, shortness of breath, and fatigue. In severe cases, symptoms can progress to acute respiratory distress syndrome and multisystem organ failure [6,7]. COVID-19 complications include thromboembolic events, hypoxemic respiratory failure, acute kidney injury, and multisystem inflammatory syndrome in children (MIS-C) [8,9]. Postacute COVID-19 syndrome, or "long COVID," involves symptoms persisting beyond four weeks after infection, such as fatigue, joint and muscle aches, chest pain, cognitive impairment, and mood changes [9].

Treatment for COVID-19 varies based on disease severity. Mild cases are managed with supportive care, including rest, hydration, and nutrition, along with antipyretics and analgesics as needed [10]. Antiviral therapies such as remdesivir have shown efficacy in inhibiting viral replication [11], while corticosteroids are reserved for severe cases due to their potential to reduce lung inflammation [12]. However, corticosteroid use is associated with serious side effects, including steroid-induced avascular necrosis (AVN) of the femoral head [13]. Consequently, WHO guidelines recommend avoiding corticosteroids for mild-to-moderate COVID-19 unless indicated for another reason [14].

AVN is characterized by ischemic injury to bone tissue, leading to bone death and joint destruction. This condition often affects the hip, with common causes including trauma, corticosteroid use, alcohol abuse, and systemic diseases such as sickle cell disease and lupus [15,16]. Early stage AVN is typically asymptomatic, while advanced stages present limited movement, pain, and swelling in the affected joint [17,18]. Diagnosis is typically confirmed through X-ray and MRI [17,19].

Management of AVN aims to prevent bone collapse, long-term disability, and chronic pain, with treatments ranging from nonoperative interventions such as pain management and physiotherapy to surgical options such as core decompression and joint replacement [17,20]. The relationship between COVID-19 and AVN has gained significant attention, with studies suggesting that COVID-19 may increase the incidence of AVN through a hypercoagulable state and the use of corticosteroids in treatment [21,22]. A history of COVID-19 diagnosis has been linked to a higher occurrence of idiopathic osteonecrosis in patients undergoing total hip arthroplasty (THA), indicating a potential role of COVID-19 in the development of osteonecrosis [23,24].

In this systematic review, we aim to explore the association between COVID-19 and AVN, extracting data from multiple studies to understand the potential impact of COVID-19 on AVN incidence and outcomes. We also evaluated the severity and outcomes related to AVN, such as pain and mobility.

## Review

Methods and materials

Searching Strategy

We conducted a systematic review according to the 2020 Preferred Reporting Items for Systematic Reviews and Meta-Analyses (PRISMA) guidelines [25]. We searched the following five databases: PubMed, Web of Science, Cochrane Library, Scopus, and Embase up to May 30, 2024. The following terms were used in our search ("COVID-19" OR "SARS-CoV-2" OR "coronavirus") AND ("Avascular Necrosis" OR "Osteonecrosis" OR "Aseptic Necrosis"). The used search query was as follows: ("COVID-19" OR "COVID 19" OR "2019-nCoV Infection" OR "2019 nCoV Infection" OR "2019-nCoV Infections" OR "Infection, 2019-nCoV" OR "SARS-CoV-2 Infection" OR "Infection, SARS-CoV-2" OR "SARS CoV 2 Infection" OR "SARS-CoV-2 Infections" OR "2019 Novel Coronavirus Disease" OR "2019 Novel Coronavirus Infection" OR "2019-nCoV Disease" OR "2019 nCoV Disease" OR "2019-nCoV Diseases" OR "Disease, 2019-nCoV" OR "COVID19" OR "Coronavirus Disease 2019" OR "Disease 2019, Coronavirus" OR "Coronavirus Disease-19" OR "Coronavirus Disease 19" OR "Severe Acute Respiratory Syndrome Coronavirus 2 Infection" OR "COVID-19 Virus Disease" OR "COVID 19 Virus Disease" OR "Disease, COVID-19 Virus" OR "Virus Disease, COVID-19" OR "SARS Coronavirus 2 Infection" OR "COVID-19 Virus Infection" OR "COVID 19 Virus Infection" OR "COVID-19 Virus Infections" OR "Infection, COVID-19 Virus" OR "Virus Infection, COVID-19" OR "COVID-19 Pandemic" OR "COVID 19 Pandemic" OR "Pandemic, COVID-19" OR "COVID-19 Pandemics") AND ("Avascular Necrosis" OR "Osteonecrosis" OR "Femur Head Necrosis" OR "Legg-Calve-Perthes Disease" OR "Osteonecroses" OR "Bone Necrosis" OR "Bone Necroses" OR "Necroses, Bone" OR "Necrosis, Bone" OR "Kienbock Disease" OR "Kienboeck Disease" OR "Kienbock's Disease" OR "Kienboecks Disease" OR "Necrosis, Avascular, of Bone" OR "Avascular Necrosis of Bone" OR "Bone Avascular Necrosis" OR "Aseptic Necrosis of Bone" OR "Bone Aseptic Necrosis").

Eligibility Criteria and Study Selection

We retrieved all the studies including COVID-19 patients and AVN disease. We also included cohorts and case series studies. RCTs were not an option due to the nature of COVID-19 studies. Case reports, posters, abstracts, editorials, comments, reviews, cross-sectional articles, and non-English articles were excluded. We also excluded any study including mucormycosis or any other bone infection associated with COVID-19. We screened the studies by title and abstract for relevance, and then a full-text review was conducted for related studies to assess their probability for inclusion in our systematic review. A third senior author established disagreements. During the full-text review, we noted two studies with the same manuscript and another two studies with the same patient demographics, and these studies were excluded.

Data Extraction

We extracted the following data to collect relevant information: (1) studies characteristics (author, year, study design, sample size, site, follow-up period, inclusion criteria, primary endpoints, aim of the study, conclusion, and limitations), (2) population details (age, sex, BMI, diabetes and hypertension prevalence, and COVID-19 severity), (3) intervention details (dose of corticosteroids per day or the cumulative dose, and duration of corticosteroids intake), and (4) outcomes (incidence of AVN, the time between the onset of COVID-19 and the AVN symptoms, bone affected by AVN, associated infection such as “septic arthritis,” orthopedic surgeries after AVN, Ficat and Arlet classification system for MRI, pre and post-treatment Harris Hip Score [HHS], visual analog scale [VAS] pain initially and at follow-up VAS).

Risk-of-Bias Assessment

To evaluate the quality of single-arm cohort studies and case series, we adapted the Newcastle-Ottawa Scale (NOS). The NOS is traditionally used to assess the quality of non-randomized studies, specifically cohort and case-control studies. For our purposes, the scale was modified to better suit the assessment of single-arm cohorts and case series. The NOS evaluates studies based on three general categories: selection (3 points), comparability of study groups (2 points), and outcome ascertainment (3 points). Studies scoring 6-7 are considered high-quality, those with scores between 4 and 5 are classified as fair quality, and those scoring below 4 are deemed poor quality.

Results

Literature Search

**Figure 1:**
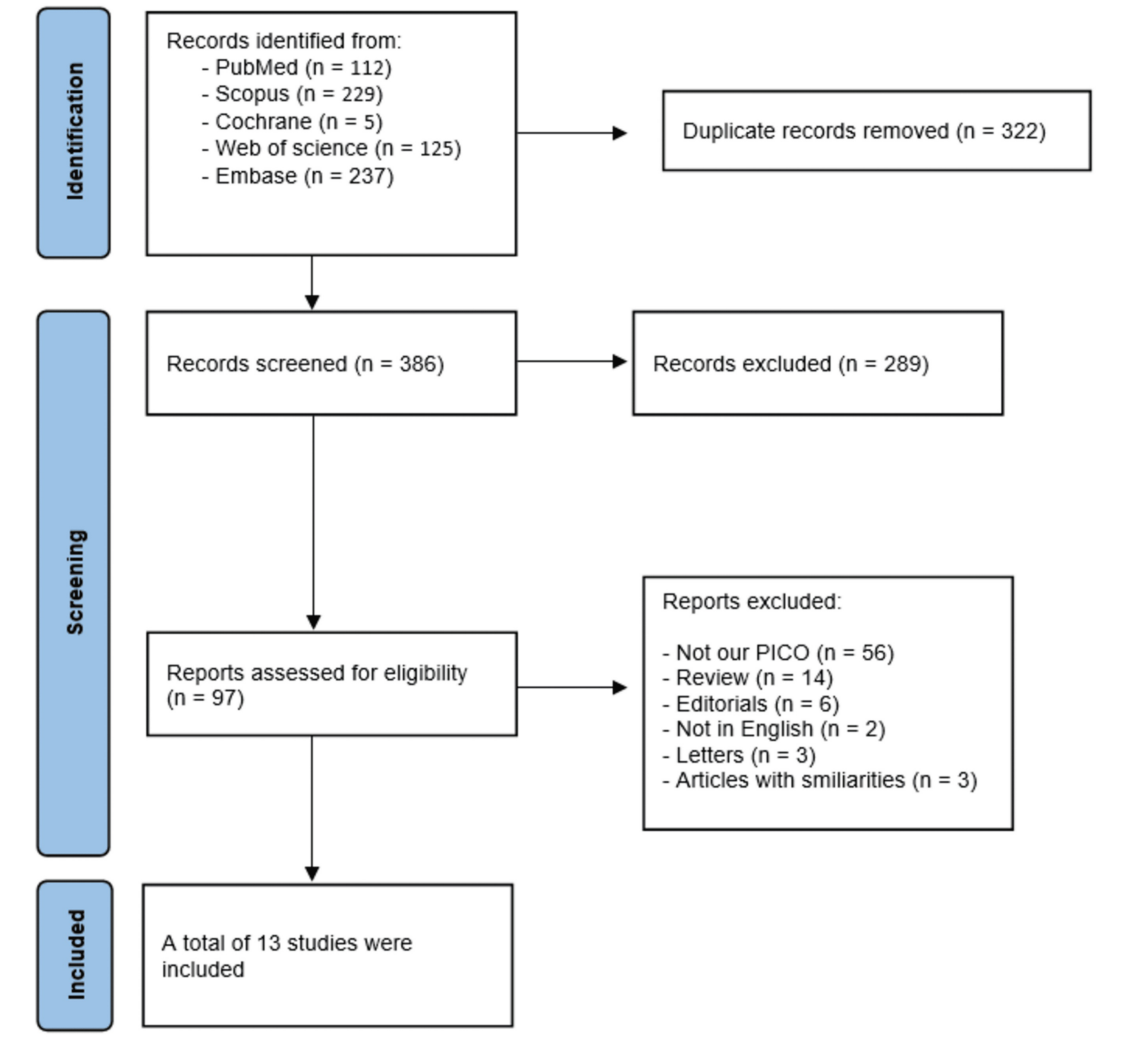
PRISMA flow diagram Studies: [23,24,26-36]

A total of 708 studies were identified during the literature search: 112 from PubMed, 229 from Scopus, 5 from Cochrane, 125 from Web of Science, and 237 from Embase. We removed 322 duplicates. This left 386 articles for screening by titles and abstracts using EndNote. Then, we reviewed 97 articles in full text to determine their eligibility. We excluded 84 studies (28 with no relevance, 26 case reports, 2 cross-sectional studies, 14 reviews, 6 editorials, 2 not in English, 3 letters, and 3 articles with similarities). Finally, we included 13 studies that fit our selection criteria [23,24,26-36]. The studies are included in a PRISMA flow diagram shown in Figure 1.

Study Characteristics

The included studies comprise nine case series and four cohort studies with varying follow-up durations. The follow-up periods ranged from a short follow-up duration of 1.5 months to a 24-month follow-up period, reflecting diverse observational timelines. Case series provide detailed accounts of individual cases and highlight specific clinical observations and outcomes related to osteonecrosis and AVN post-COVID-19. Cohort studies offer broader perspectives by following groups of patients over time to assess the incidence and progression of these conditions. Our included studies had high variations in the corticosteroid treatment protocols due to each patient's evolving nature and severity of COVID-19 symptoms. Study details are shown in Table 1.

**Table 1:** Summary of the included studies Studies included: [23,24,26-36] AVN, avascular necrosis; CS, corticosteroids; ICD-10, International Classification of Diseases, 10th Revision; n/a, not available; ONC, osteonecrosis; ONFH, osteonecrosis of the femoral head; PC-RONJ, post-COVID-related osteonecrosis of the jaw; PCR, polymerase chain reaction; SA, septic arthritis

Study	Study design	Country	Follow-up period (months)	Inclusion criteria	Purpose	Conclusion
Agarwala et al., 2022 [23]	Case series	India	3	n/a	The researchers highlight the critical need for prompt diagnosis through MRI imaging to detect ONC in its initial stages, allowing for timely intervention with bisphosphonate treatment. Their collection of cases seeks to inform medical practitioners about this possible enduring consequence of COVID-19 infection and emphasizes the significance of quick identification and therapeutic management to improve patient outcomes.	CS treatments have proven crucial for saving lives during the COVID-19 crisis, though evidence suggests that patients infected with COVID-19 develop ONC more frequently, more rapidly, and with smaller steroid doses than typically expected. Knee ONC, when identified early, can be effectively treated using bisphosphonate medications. Therefore, when patients who have recovered from COVID-19 present with knee problems, prompt MRI evaluation based on clinical suspicion is recommended to facilitate timely bisphosphonate intervention and improve outcomes.
Al-Mahalawy et al., 2022 [33]	Case series	Egypt	2	Patients burdened with maxillary ONC behavior with a history of a recent SARS-CoV-2 infection according to the PROCESS guideline.	This series reports the growing number of ONC cases associated with post‑COVID‑19 patients.	Emerging evidence suggests that PC-RONJ may now be classified as one of the possible oral and maxillofacial complications following COVID-19 infection, with cases occurring spontaneously and predominantly affecting the upper jaw (maxilla) rather than being triggered by specific events or treatments.
Ardakani et al., 2022 [26]	Case series	Iran	3	n/a	The simultaneous occurrence of femoral head AVN and SA as a complication following COVID-19 infection has not previously been reported in medical literature. Given the widespread administration of potentially life-saving CS treatments in COVID-19 patients, this report serves as a cautionary alert to clinicians regarding the possibility of hip joint infections developing in this patient population.	For COVID-19 patients with a history of corticosteroid treatment during their illness, clinicians should maintain heightened vigilance regarding any joint symptoms, particularly those affecting the hip area, as these may indicate underlying joint infection. Prompt recognition and surgical intervention when needed can be crucial for preserving hip joint function and preventing long-term complications that might otherwise lead to permanent joint damage.
Assad et al., 2023 [32]	Case series	Iraq	24	All patients with a prior history of COVID-19 who developed AVN.	The current study aims to report several cases of AVN after being infected with SARS-CoV-2.	The COVID-19 pandemic continues to reveal various long-term health consequences beyond the initial infection, with AVN emerging as a potential delayed complication that clinicians should monitor. Identifying AVN in its early stages is particularly crucial, as prompt initiation of appropriate treatment protocols can effectively prevent the progression to bone collapse and associated permanent structural damage that would otherwise require more invasive interventions.
Dhanasekararaja et al., 2022 [24]	Prospective cohort	India	n/a	Patients recovered from COVID-19 and were diagnosed with ONFH from November 2020 to October 2021.	We aim to report the consecutive patients diagnosed with ONFH following recovery from COVID-19 disease and elucidate the unique features of ONFH associated with COVID-19.	COVID-19-related ONFH presents with varying clinical manifestations, ranging from typical ONFH patterns to more severe cases characterized by rapid joint deterioration. These aggressive presentations often include elevated inflammatory markers in blood tests and widespread edema affecting the surrounding bone and soft tissues near the joint. The observation that ONFH developed despite relatively low cumulative steroid doses in these patients suggests that the vasculitis associated with COVID-19 infection likely contributes significantly to the underlying disease mechanism rather than steroid therapy alone being responsible.
Kachewar and Kachewar, 2022 [30]	Retrospective cohort	India	n/a	a. Positive RT-PCR for SARS-CoV-2 obtained with nasopharyngeal/oropharyngeal swabs or COVID-19 positive on RAPID antigen test before starting treatment for COVID-19. b. Received steroids during the treatment for COVID-19. c. New-onset hip pain was not there before being affected by COVID-19. d. MRI scan is done.	The aim is to analyze the spectrum of AVN of the femoral head as seen in MRI images of patients treated for COVID-19.	The study found AVN developing in 6% of patients who received standard COVID-19 treatment and subsequently experienced hip pain. This complication appeared more frequently in younger individuals under 40 years old, likely because they resumed physical activities and regular work more quickly after recovery. When these post-COVID hip pain cases underwent MRI evaluation, most actually showed normal findings without evidence of pathology. Among those with positive MRI results, most displayed only mild stage I AVN changes, suggesting early disease that might respond well to intervention.
Kandari et al., 2022 [34]	Case series	India	7	Patients were confirmed COVID-19 positive with nucleic acid testing in a time frame from April 2020 to September 2020	The primary endpoints of this case series are: (1) to highlight the potential link between CS treatment for COVID-19 and ONC of the ONFH and (2) to emphasize the importance of early detection of ONFH in patients with a history of COVID-19 who received CSs.	Building on our clinical observations, we found substantial reason to suspect that ONFH may develop in patients who received CS therapy following COVID-19 diagnosis. When managing these cases, professionals should consider the documented experiences from the earlier SARS outbreak as valuable guidance given the still-developing evidence base for COVID-19-related complications. The findings establish preliminary groundwork that should encourage further research investigations focused on this specific relationship between COVID-19, steroid treatment, and femoral head ONC.
Khan et al., 2023 [35]	Case series	India	7.7	Case records were reviewed of all the jaw ONC patients treated at our center from April 2021 till March 2023 with a history of severe COVID-19.	To ascertain the correlation between COVID-19 infection and jaw ONC, along with identifying risk factors that could be associated with the development of the condition. Another aim of our study is to establish whether maxillofacial ONC is an early or late complication seen in COVID-19 patients.	The development of jaw ONC may result from a combination of three key factors: blood clotting abnormalities following COVID-19 infection, steroid treatment received during COVID-19 management, and subsequent dental procedures that trigger the bone condition. Notably, this can affect individuals without any prior systemic health conditions and may manifest nearly two years after the initial COVID-19 infection, suggesting a need for extended vigilance in post-COVID dental care.
Jha and Sidhu, 2024 [28]	Case series	India	7	Patients hospitalized during active COVID disease from 2020 to 2022 and those who complained of hip disorders, PCR indicating positive COVID-19 infection, and joint pain during the course of the disease and follow-up.	To analyze the effects of COVID-19 disease on the hip bone and joint tissue.	SAR-CoV-2 can affect bones, presenting with symptoms 2–3 weeks after infection. This may resolve with medical management or result in end-stage AVN that may respond well to core decompression or hip arthroplasty. The effects of COVID-19 infection on the human body are complex, including the musculoskeletal system. Furthermore, long-term studies are suggested to have a better understanding of the disease.
Panin et al., 2022 [27]	Case series	Russia	n/a	n/a	Possible link between COVID-19 and accelerated development of ONC	The researchers examined potential connections between COVID-19 infection and the development of ONC. Although CSs used in COVID-19 treatment protocols likely contribute to this bone complication, the researchers noted that ONC appeared more rapidly than typically observed in standard steroid-induced cases, indicating that additional COVID-specific mechanisms may be involved in the pathological process. Their investigation also considered alternative non-steroid pathways and genetic susceptibility factors that might predispose specific individuals to develop this condition. The authors stress that additional comprehensive studies are necessary to fully clarify and establish the specific relationship between COVID-19 and the occurrence of ONC.
Parikh et al., 2023 [36]	Case series	USA	1.5	n/a	The primary objective of this study is to establish if AVN presents as an enduring sequela among COVID-19 survivors, alongside examining the interplay between AVN occurrence, the underlying disease mechanisms of COVID-19, and the steroid therapies frequently used in COVID-19 treatment protocols—all to determine whether these elements contribute separately or in combination to elevate the probability of bone tissue death.	Research suggests COVID-19 infection combined with CS treatment may significantly increase AVN risk even in otherwise healthy patients. Clinicians should use caution when prescribing steroids for COVID-19 cases, carefully managing dosages based on individual factors. A risk stratification system considering both disease severity and steroid exposure could identify patients needing closer monitoring. Regular hip pain assessment during follow-ups and increased vigilance would enable earlier AVN detection, potentially preventing permanent joint damage.
Veizi et al., 2023 [29]	Prospective cohort	Turkey	24	Age between 18 and 60 years, hospitalization at our center with ICD-10 codes related to COVID-19 (U06, U07.0, U07.1, and U07.2) for at least five days, a positive PCR result confirming the COVID-19 diagnosis and no prior history of CS use.	To evaluate the incidence of ONC, with a special focus on ONFH, in novel COVID-19 patients two years after the pandemic.	COVID-19 patients treated with CSs are showing an increasing rate of ONC, with approximately 5% developing this complication within two years of treatment. Persistent joint pain remains a common complaint among COVID-19 survivors, even after recovering from the primary infection. Interestingly, current data show no clear correlation of ONC development with treatment duration, total CS dose, or maximum single-day dosage. Additional research with larger patient populations is necessary to establish more definitive conclusions regarding this emerging post-COVID complication.
Velchov et al., 2023 [31]	Retrospective cohort	Bulgaria	6	n/a	Explore the incidence of femoral head AVN associated with CS therapy in 24 patients diagnosed with severe COVID-19 at a single center.	Research results from this investigation confirm earlier studies and clinical case reports indicating a significant rise in femoral head AVN cases throughout the COVID-19 pandemic, attributed to the administration of high-dose CS regimens in patients who required hospitalization for severe COVID-19 pneumonia.

Population

Our systematic review included a total of 795 patients diagnosed with COVID-19. The mean age of patients ranged from a mean of 34 years up to 58.8 years, with a higher prevalence (around 66%) of male patients. The mean BMI values ranged from 25 kg/m² to 34.3 kg/m². The prevalence of HTN ranged from 8.5% to 46.1%, with an average of 12.81% across the total population. Regarding the use of corticosteroids, it can be observed that the majority of patients in the included studies used corticosteroids, except for a few studies where a significant percentage of patients did not receive corticosteroids: Jha and Sidhu [28] reported that 60% of their patients did not use corticosteroids, and the control group of Veizi et al. [29] reported 100% of patients without corticosteroid use.

The severity of COVID-19 among patients was reported using various criteria. In the study by Panin et al., 25% of patients were classified as severe and 75% as moderate based on lung damage [27]. Jha and Sidhu used a 4-point scale and determined 30% of patients as severe, 50% as moderate, and 20% as mild [28]. Velchov et al. [31] determined 66.6% of patients as severe and 33.3% as moderate according to their oxygen requirements. Kandari et al. [34] classified 9% of patients as severe and 91% as moderate based on ICU admissions. In Khan et al. [35], all patients were categorized as severe according to their medical history. More details of patients' demographics are shown in Table 2.

**Table 2:** Demographic data of patients involved in the studies References: [23,24,26-36] HTN, hypertension; DM, diabetes mellitus; n/a, not available

Study	Sample size, n	Age (years), mean (SD)	Male, n (%)	BMI (kg/m^2^), mean (SD)	DM, n (%)	HTN, n (%)	Patients not using corticosteroids (%)	COVID-19 severity (%)
Agarwala et al., 2022 [23]	2	n/a	1 (50)	n/a	n/a	n/a	0	n/a
Al-Mahalawy et al., 2022 [33]	12	56.1 (9.65)	5 (41.7)	n/a	12 (100)	5 (41.7)	0	n/a
Ardakani et al., 2022 [26]	5	38.4 (16.15)	2 (40)	n/a	n/a	n/a	0	n/a
Assad et al. 2023 [32]	17	38.65 (6.1)	12 (70.6)	28.3 (2.4)	n/a	n/a	3 (17.6)	n/a
Dhanasekararaja et al., 2022 [24]	22	38.82 (14.03)	20 (90.9)	27.54 (4.39)	2 (9.09)	4 (18.18)	0	n/a
Kachewar and Kachewar, 2022 [30]	200	45 (12.5)	154 (77)	n/a	12 (6)	17 (8.5)	0	n/a
Kandari et al., 2022 [34]	11	46 (8.35)	9 (81.8)	n/a	n/a	1 (9)	0	Severe in 30, moderate in 50, mild in 20
Khan et al., 2023 [35]	13	47.4 (16.8)	8 (61.5)	n/a	6 (46.1)	6 (46.1)	0	Severe in 9, moderate in 91
Jha and Sidhu, 2024 [28]	10	58.8 (11.3)	4 (40)	n/a	2 (20)	1 (10)	60	Severe in 100
Panin et al., 2022 [27]	4	34 (4.7)	2 (50)	n/a	n/a	n/a	0	Severe in 25, moderate in 75
Parikh et al., 2023 [36]	3	55.6 (10.8)	2 (66.6)	n/a	n/a	1 (33.3)	0	n/a
Veizi et al., 2023, control group [29]	236	42.8 (12.2)	133 (56.4)	n/a	Excluded	n/a	100	n/a
Veizi et al., 2023, corticosteroids group [29]	236	41.7 (11.8)	156 (66.1)	n/a	Excluded	n/a	0	n/a
Velchov et al., 2023 [31]	24	56 (15)	17 (70.8)	34.3 (2)	n/a	n/a	0	Severe in 66.6, moderate in 33.3

Intervention

Among the included studies, the cumulative dose of steroids varied widely, with averages ranging from approximately 811.08 mg to 1,863.6 mg, with an average cumulative dose of 1,462.9 mg. The daily dose of steroids is reported in a few studies (n=3). Two of them [28,33] reported a dose of 6 mg/day, and the maximum dose per day was reported by Velchov et al. [31], which was 60.6 mg. The duration between COVID-19 infection and initial bone symptoms showed noticeable variability, ranging from 2 to 62 weeks, with an average of 17.5 weeks. Duration of steroid intake ranged from 5 days up to 10 weeks, with an average duration of 2.81 weeks. Velchov et al. highlighted an average duration of steroid intake of approximately 2.85 weeks [31]. Full details about corticosteroids are given in Table 3.

**Table 3:** Corticosteroid intervention details References: [23,24,26-36] n/a, not available

Study	Cumulative dose of corticosteroids (mg), mean (SD)	Dose of corticosteroids mg/day, mean (SD)	Duration between COVID-19 infection and initial bone symptoms (days), mean (SD)	Duration of corticosteroids intake (weeks), mean (SD)
Agarwala et al., 2022 [23]	1,156.5 (128.25)	n/a	72.5 (47.5)	2.4 (0.28)
Al-Mahalawy et al., 2022 [33]	n/a	6	38.5 (16.32)	10
Ardakani et al., 2022 [26]	1,695.2 (158.75)	n/a	41.6 (10.7)	n/a
Assad et al. 2023 [32]	n/a	n/a	203.8 (143.57)	0.71 (0.23)
Dhanasekararaja et al., 2022 [24]	811.08 (527.3)	n/a	39.36 (22.44)	2.86 (1.08)
Kachewar and Kachewar, 2022 [30]	n/a	n/a	n/a	n/a
Kandari et al., 2022 [34]	1,863.6 (55.2)	n/a	210	3.14
Khan et al., 2023 [35]	n/a	n/a	436 (130)	n/a
Jha and Sidhu, 2024 [28]	n/a	6	14	2
Panin et al., 2022 [27]	1,759.67 (1,586.54)	n/a	113.75 (42)	n/a
Parikh et al., 2023 [36]	n/a	n/a	n/a	2.8
Veizi et al., 2023, corticosteroid group [29]	1491.9 (2506.6)	n/a	n/a	0.97 (0.58)
Velchov et al., 2023 [31]	n/a	60.6 (47.04)	57.13 (6.64)	2.85 (0.32)

Outcomes

The hip was the most affected bone in the population, followed by the femoral head, the maxilla, the knee, and the mandible. In the study by Parikh et al., one patient was reported to have AVN in multiple sites: the femur, the knee, and the hip [36]. The Ficat and Arlet classification system classifies MRI findings into five stages: stage 0, where the MRI is normal; stage I, showing bone marrow edema; stage II, displaying a geographic defect, with stage IIA showing mild subchondral collapse and stage IIB demonstrating more extensive subchondral collapse. Stage III is characterized by the crescent sign and cortical collapse, and stage IV indicates secondary degenerative changes with joint space narrowing and acetabular involvement. The classification was applied to 267 patients, with stage II being the most common.

THA was performed on 31 hips, core decompression was performed on 15 hips, and surgical debridement was performed on 25 patients affected with AVN of the maxilla and the mandible. Pre-treatment and post-treatment HHS were used in several studies (n=5). Assad et al. showed an increase from a mean pre-treatment score of 63.64 to a post-treatment score of 82.64 [32]. Ardakani et al. reported an initial VAS pain score of 9.4, which decreased to 2.8 after the intervention, illustrating effective pain management [26]. Jha and Sidhu [28] and Panin et al. [27] showed similar trends, with significant pain reduction post-treatment. For a more comprehensive look, refer to Table 4.

**Table 4:** Outcomes of AVN Studies references [23,24,26-36] AVN, avascular necrosis; n/a, not available; THA, total hip arthroplasty; VAS, visual analog scale

Study ID	Bone affected by AVN	Ficat and Arlet classification system for MRI (%)	Treated with orthopedic surgery (%)	Pre-treatment Harris Hip Score, mean (SD)	post-treatment Harris Hip Score, mean (SD)	VAS pain initially	Pain at follow-up VAS
Agarwala et al., 2022 [23]	Knee and hip	Stage III (50)	0	n/a	n/a	8	2
Al-Mahalawy et al., 2022 [33]	Maxilla	n/a	Surgical debridement (100)	n/a	n/a	n/a	n/a
Ardakani et al., 2022 [26]	Hip and femoral head	Stage II (20), stage IV (20)	Hip arthroplasty (100)	n/a	n/a	9.4	2.8
Assad et al. 2023 [32]	Femoral head	n/a	Core decompression (29.4)	63.64 (22.64)	82.64 (9.37)	n/a	n/a
Dhanasekararaja et al., 2022 [24]	Hip	Stage IIA (69.2), stage IIB (15.3), stage III (15.3)	THA (9.09)	63.6 (23.2)	82.6 (9.6)	n/a	n/a
Kachewar and Kachewar, 2022 [30]	Hip	Stage I (3), stage II (2), stage III (1)	0	n/a	n/a	n/a	n/a
Kandari et al., 2022 [34]	Femoral head	Stage I (6.25), stage II (50), stage III (31.25), stage IV (12.5)	THA (18.18), core decompression (9.09)	62	76	7	3
Khan et al., 2023 [35]	Maxilla and mandible	n/a	Surgical debridement (100)	59.2 (11.4)	86.8 (7.2)	n/a	n/a
Jha and Sidhu, 2024 [28]	Hip	n/a	Core decompression (40)	n/a	n/a	8	0.5
Panin et al., 2022 [27]	Femoral head	n/a	THA, (25)	n/a	n/a	8	4
Parikh et al., 2023 [36]	Hip, femur, and knee	Stage II (66.6)	Core decompression (33.3)	n/a	n/a	n/a	n/a
Veizi et al., 2023, control group [29]	Hip	n/a	n/a	n/a	n/a	n/a	n/a
Veizi et al., 2023, corticosteroid group [29]	Hip and knee	n/a	Hip replacement (0.4)	n/a	n/a	n/a	n/a
Velchov et al., 2023 [31]	Hip	Stage II (8.3), stage III (29.1), stage IV (62.5)	THA 82, core decompression (18)	59.2 (11.4)	86.8 (7.2)	n/a	n/a

Quality of the Included Studies

The NOS quality assessment revealed diverse methodological strengths and weaknesses among the 13 included studies. Eight studies, classified as good quality, demonstrated robust methodologies, including well-defined cohorts, adequate exposure and outcome ascertainment, and sufficient follow-up durations. These studies will likely provide more reliable insights into the association between COVID-19 and AVN. One study received fair quality ratings, indicating moderate reliability due to some methodological concerns in the selection domain. Three studies, rated as poor, exhibited significant methodological flaws, including not being able to be compared based on the design or analysis adjusted for confounding factors (Table 5).

**Table 5:** Newcastle-Ottawa Scale assessment Studies: [23,24,26-36] *Asterisk indicates that the study met the Newcastle-Ottawa Scale criteria and was awarded a star for this domain. Q1, representativeness of the exposed cohort; Q2, selection of non-exposure cohort; Q3, ascertainment of exposure; Q4, demonstration that outcome of interest was not present at the start of the study; Q5, comparability of the cohort based on the design or analysis; Q6, assessment of outcome; Q7, was follow-up long enough for outcomes to occur; Q8, adequacy of follow-up of cohorts.

Study	Newcastle-Ottawa scale assessment								
	Selection				Comparability	Outcome			Quality score
	Q1	Q2	Q3	Q4	Q5	Q6	Q7	Q8	
Agarwala et al., 2022 [23]	*	-	*	*	-	-	*	*	Good
Al-Mahalawy et al., 2022 [33]	*	-	*	*	*	*	*	*	Good
Ardakani et al., 2022 [26]	*	-	*	*	*	*	-	*	Good
Assad et al. 2023 [32]	*	-	*	*	*	*	-	*	Good
Dhanasekararaja et al., 2022 [24]	*	-	*	*	-	*	*	*	Poor
Kachewar and Kachewar, 2022 [30]	-	-	*	*	-	*	-	*	Poor
Kandari et al., 2022 [34]	*	-	-	*	*	*	*	*	Fair
Khan et al., 2023 [35]	*	-	*	*	*	*	*	*	Good
Jha and Sidhu, 2024 [28]	*	-	*	*	*	*	*	*	Good
Panin et al., 2022 [27]	*	-	*	*	-	*	*	*	Poor
Parikh et al., 2023 [36]	*	-	*	*	-	*	*	-	Poor
Veizi et al., 2023 [29]	*	*	*	*	*	*	*	*	Good
Velchov et al., 2023 [31]	*	-	*	*	*	*	-	*	Good

Discussion

This systematic review comprehensively examined the association between COVID-19 infection and the development of AVN across 13 relevant studies involving 795 patients. The severity of COVID-19 varied, with a substantial proportion experiencing severe manifestations, necessitating intensive care and prolonged hospital stays. Corticosteroid therapy, commonly used in severe COVID-19 cases, further heightens this risk due to its known association with osteonecrosis development, especially at higher cumulative doses and longer durations, as shown in our included studies [18,37,38].

The duration from COVID-19 infection to initial bone symptoms was an average of 17.5 weeks; however, Karpur et al. highlighted that the mean interval between the onset of initial symptoms and MRI for AVN of the hip joint in COVID-19 patients was two to four weeks, indicating a relatively short timeframe for the manifestation of bone-related issues post-COVID-19 infection [39]. The most common stage of AVN is stage II, which is different from previous studies suggesting stage I is the most prevalent [39, 40]. These findings emphasize the critical role of early detection and intervention in managing AVN among COVID-19 survivors. Orthopedic interventions such as THA and core decompression surgery (CDS) have improved functional outcomes and reduced AVN pain [23,24,28,29,31,33]. Moreover, tools such as the HHS and VAS for pain assessment provide standardized measures for evaluating treatment efficacy and patient outcomes post-intervention [23,24,30,32,35,36].

Surgical management is essential, especially in late cases. Studies indicate that COVID-19 survivors with femoral head AVN often require surgical interventions such as THA or CDS [39, 40]. These findings underscore the importance of early intervention and tailored treatment strategies in managing AVN. Dhanasekararaja et al. [24] divided the study population into two categories: osteonecrosis of the femoral head (ONFH) and rapidly destructive coxarthrosis (RDC). RDC, a rare condition typically affecting the elderly (60-66 years) [41], was also observed in patients over 50 years in their study. RDC involves chondrolysis, which is rapid and diffuse destruction of cartilage leading to the narrowing or loss of joint space, ultimately resulting in femoral head destruction within a year [41]. This condition can be associated with either ONFH or osteoarthritis [42].

The same study classified the groups based on MRI findings, hip aspiration, and inflammatory markers such as C-reactive protein (CRP) [24], noting fluctuating CRP levels in some cases. Clinical signs of RDC include hip pain and rapid femoral head destruction with joint damage [43]. Moreover, Dhanasekararaja et al. [24] reported a significant elevation of CRP in the RDC group compared to classic ONFH, demonstrating an aggressive presentation with extensive periarticular bone and soft tissue edema. They recommended hip aspiration to differentiate RDC from primary septic arthritis of the hip, as RDC often necessitates THA [24,43].

The incidence of osteonecrosis in COVID-19 patients has shown an increase. Studies have highlighted this association, with findings indicating a rise in osteonecrosis cases during the pandemic period [44]. Veizi et al. revealed a higher incidence in those receiving corticosteroid treatment, emphasizing the impact of COVID-19 on osteonecrosis development [29].

Our review corroborates previous studies suggesting an increased risk of AVN associated with COVID-19 infection, potentially exacerbated by hypercoagulability induced by the virus and the use of corticosteroids [45,46]. The pathophysiological mechanisms linking COVID-19 to AVN include thrombotic events secondary to systemic inflammation and hypoxia, which compromise blood flow to bones, thereby predisposing patients to osteonecrosis [47,48]. In COVID-19, cytokines play a crucial role in the inflammatory response, with elevated levels of interleukin (IL)-6 being associated with disease severity and prognosis [49, 50]. The dysregulation of cytokines, including TNF-α, IL-1β, and IL-6, can lead to a cytokine storm, contributing to the pathophysiology of COVID-19 and potentially causing severe inflammation and poor outcomes [51].

The present systematic review demonstrates several methodological strengths, particularly in its multifaceted approach to topic coverage and its broad data acquisition strategy, which collectively enabled the generation of comprehensive research findings. However, the review also has limitations. There was no clear comparator arm due to the nature of the study as the results were mainly obtained retrospectively. The included studies exhibit significant heterogeneity in patient demographics, corticosteroid treatment protocols, and follow-up durations, which may affect the generalizability of our conclusions. Furthermore, the observational nature of most studies and the reliance on self-reported data introduce potential biases.

The findings of this review have important implications for clinical practice and health policy. Healthcare providers should be aware of the potential risk of AVN in COVID-19 patients, particularly those treated with corticosteroids. This awareness should translate into more vigilant monitoring and timely interventions to prevent severe outcomes. Regular monitoring for early signs of AVN should be integrated into the follow-up care of COVID-19 survivors. Incorporating routine imaging studies, such as MRI, may facilitate early detection and intervention, potentially mitigating the severity of AVN. Future research should focus on well-designed prospective studies with a clear control group for comparison or other direct comparison between different treatment approaches.

## Conclusions

This systematic review underscores the association between COVID-19 infection, corticosteroid use, and the development of AVN. The findings highlight the need for careful consideration in prescribing corticosteroids to COVID-19 patients and underscore the importance of early detection and intervention in managing AVN. Despite the limitations of the current evidence, this review provides valuable insights for clinical practice. It emphasizes the urgency of further research to enhance our understanding and management of AVN in the context of COVID-19.
